# Antibacterial Activity of Essential Oils and *Trametes versicolor* Extract against *Clavibacter michiganensis* subsp. *michiganensis* and *Ralstonia* *solanacearum* for Seed Treatment and Development of a Rapid In Vivo Assay

**DOI:** 10.3390/antibiotics9090628

**Published:** 2020-09-21

**Authors:** Laura Orzali, Maria Teresa Valente, Valeria Scala, Stefania Loreti, Nicoletta Pucci

**Affiliations:** CREA-DC Research Centre for Plant Protection and Certification, via C.G. Bertero, 22, 00156 Rome, Italy; laura.orzali@crea.gov.it (L.O.); mariateresa.valente@crea.gov.it (M.T.V.); valeria.scala@crea.gov.it (V.S.); stefania.loreti@crea.gov.it (S.L.)

**Keywords:** *Solanum lycopersicum*, tomato seed treatment, antibacterial activity, phytotoxicity, rapid molecular screening

## Abstract

*Clavibacter michiganensis* subsp. *michiganensis* (Smith) Davis et al. (Cmm) and *Ralstonia solanacearum* Yabuuchi et al. (Smith) (Rs) are important seed-borne bacterial pathogens of tomato (*Solanum lycopersicum*) listed as A2 pests in the EPPO (European and Mediterranean Plant Protection Organization) region. At present, there are few strategies to control these pathogens, and seed control with eco-compatible approaches is widely encouraged. In this work, the essential oils (EOs) of oregano (*Origanum vulgare*), garlic (*Allium sativum*), basil (*Ocimum basilicum*), cinnamon (*Cinnamomum zeylanicum*), clove buds (*Syzygium aromaticum*), thyme (*Thymus vulgaris*), and *Trametes versicolor* extract (Tve) were tested in vitro for their antimicrobial activity against Cmm and Rs (broth microdilution method). The tested EOs and the Tve extract caused a significant inhibition of bacterial growth, with very promising MBC (minimum bactericidal concentration) and MIC90 (minimum inhibitory concentration causing a 90% growth inhibition) values. Moreover, an in vivo germination test showed no major reduction in seed germination when the substances were applied as seed treatment. A rapid molecular screening method has been developed, through real-time PCR, for the specific quantification of Cmm in the presence of a vegetable matrix to test in vivo the antimicrobial efficacy of oregano and cinnamon oil on seed treatment without resorting to whole plant essays, which are time- and space-consuming.

## 1. Introduction

*Clavibacter michiganensis* subsp. *michiganensis* (Smith) Davis, Gillaspies, Vidaver & Harris (Cmm) and *Ralstonia solanacearum* (Smith) Yabuuchi et al. (Rs) are important bacterial pathogens of tomato (*Solanum lycopersicum* L.), able to cause economic losses worldwide. Cmm, the seed-borne Gram-positive bacterium that is the causal agent of bacterial canker, is considered to be one of the most destructive and economically significant diseases of tomato [1] in temperate climates and greenhouses worldwide. The disease symptoms observed in plants infected by Cmm are wilt, yellow-brown vascular discoloration, cankers on stems, and petioles and lesions on fruit [2]. Cmm is transmitted to flowers and settles internally in developing seeds through vascular tissues [2], but also externally through tomato fruit lesions [3]; moreover, it may contaminate the outside of the seed coat [4]. It is reported that one infected seed in 10,000 is able to establish an epidemic [2,5], and a population of 10^2^ CFU (colony forming units)/seed has been suggested as the probable threshold level for pathogen transmission from naturally infected seeds [6]. Latently infected young plants or asymptomatic infections during late stages of plant development, resulting in the production of contaminated seeds, are also considered important for disease spread [7,8,9]. Prevention, based on seed testing and maintaining pathogen-free seeds and transplants, is currently the most appropriate approach for disease control, and cultural practices such as eradication of infected plants and disinfestation of materials are recommended [2,7,10]. Zero tolerance import and export restrictions have been implemented for Cmm in the European Union with the Commission Implementing Regulation (EU) 2019/2072 of 28 November 2019.

Bacterial wilt, a xylem disease causing vascular dysfunction, is caused by Rs, a Gram-negative plant pathogenic bacterium infecting a large range of economically important hosts. The symptomatology in tomato includes wilting of upper leaves, development of adventitious roots along the stem, and wilting of the whole plant in hot humid environmental conditions. A longitudinal section of the stem reveals the presence of a brown discoloration of vascular tissue with leaking of drops of bacterial exudate. Rs can survive for a long time in the soil and infect the plant through wounds; dissemination can also occur through infected propagation material and water. On the basis of scientific and economic impact, Rs is ranked second in the list of top ten phytobacteria [11] and was classified as a bioterrorism agent in the USA in 2002 [12]. In tomato, its ability to spread through the seed is controversial [13,14], although, recently, Dey et al. [15] highlighted the transmissibility of Rs in tomato and brinjal seeds, confirming previous evidence [16,17] and thus assuming its economic importance for seed trade and exchange.

Both bacteria are listed as A2 pests by the European and Mediterranean Plant Protection Organization (EPPO).

Seed-borne bacterial diseases are a serious concern in conventional as well as organic/low input crop systems and represent a very critical issue for a successful production. The use of untreated seed infected with seed-borne pathogens can have a direct effect on yield and on the spread of pathogen inoculum in the soil, leading to the introduction of diseases into previously unaffected areas or their re-emergence [18]. Seed treatment with plant protection products represents one of the first options useful to reduce seed infection or contamination and prevent pathogen dissemination. Recently, the European Union has been supporting several initiatives in favor of sustainable agriculture enclosed in the New Common European Agricultural Policy 2014–2020 (CAP). According to these community initiatives, many of the plant protection products currently in use will need to be replaced by substances with a lower environmental impact (implementing Regulation (EU) 2015/408). For this reason, many scientific works have focused on developing alternative environmentally friendly measures to the use of pesticides for managing crop diseases [19,20,21,22]. A very promising approach consists in the use of natural compounds such as plant or fungal extracts and their active principles (alkaloids, phenols, monoterpenes and sesquiterpenes, isoprenoids), which have been studied for their various antimicrobic and antioxidant properties [23,24,25,26,27]. EOs (essential oils) are secondary metabolites accumulated by aromatic or medical plants and extracted from leaves, flowers, roots, and barks. The action of EOs for plant pest control and their possible use in agriculture are well documented [21,26,28,29,30]. In particular, the action of EOs for gram-positive and gram-negative plant pathogenic bacteria has been reported from 1963 [31,32,33,34,35,36,37]. The edible and non-toxic basidiomycete *Trametes versicolor* extract (Tve) is able to produce bioactive substances [38,39], in particular esopolysaccharides and glycoprotein fractions. Several studies demonstrated the bioactivity of mushroom compounds as therapeutic tools [40,41], while few studies concerned the control of plant diseases. The bioactive compounds present in Tve cultural filtrates were studied in depth against the cereal pathogenic fungi and mycotoxigenic fungi [27,42,43]. Moreover tramesan, the purified polysaccharide fractions, was effective in the control of the septoria leaf blotch complex (SLBC) by eliciting durum wheat innate defense.

In this work, the EOs of oregano (*Origanum vulgare*), garlic (*Allium sativum*), basil (*Ocimum basilicum*), cinnamon (*Cinnamomum zeylanicum*), clove buds (*Syzygium aromaticum*), thyme (*Thymus vulgaris*), and Tve were tested in vitro for their antimicrobial activity against Cmm and Rs and their potential phytotoxicity for tomato seeds. These compounds were selected on the basis of results obtained in previous papers concerning other phytopathogenic bacteria [37] and fungi [38,39]. Besides, a rapid in vivo screening test was developed (based on use of real-time PCR for the specific quantification of Cmm and Rs in the vegetable matrix) to test the antimicrobial efficacy of EOs for seed treatment, without resorting to whole plant essays, which are time- and space-consuming.

## 2. Results

### 2.1. Antibacterial In Vitro Activity

**Figure 1:**
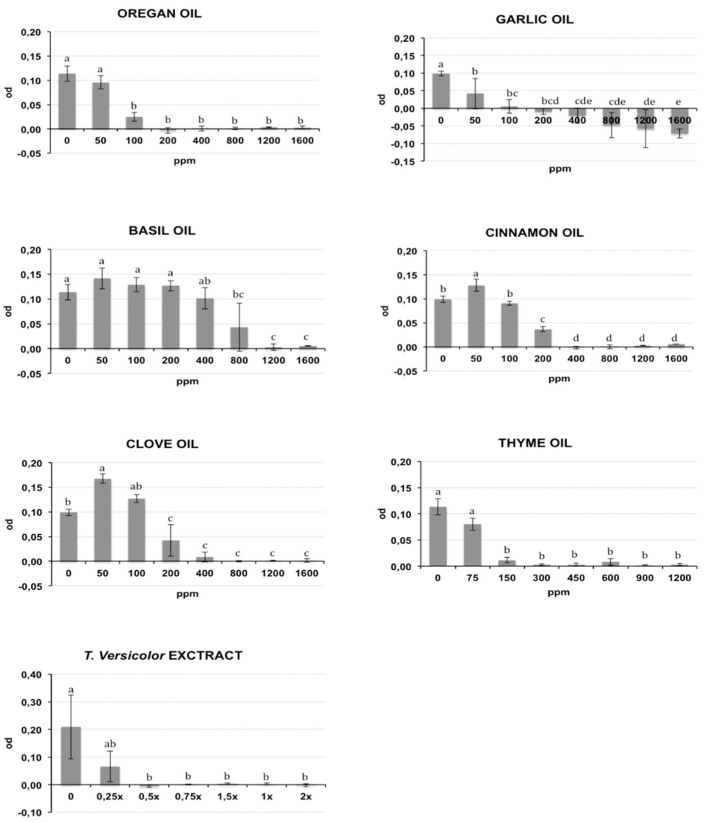
Effects of different concentrations of essential oils on *Ralstonia solanacearum* (Rs) growth measured as optical density (OD) (λ = 620 nm). The concentrations are expressed in ppm. Data reported are the means of two repetitions of the experiment. The error bar shows the standard deviation. Significant differences (*p* ≤ 0.01) found using the Tukey-Kramer test for multiple comparison are indicated as letters a–f: the same letter within the same graph means no statistical significance between the treatments.

**Figure 2:**
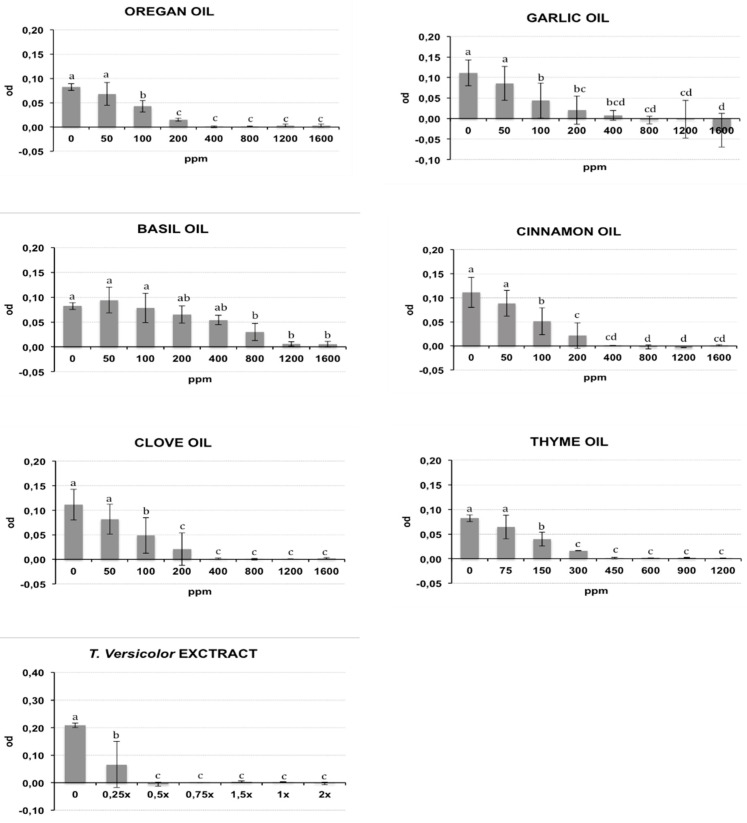
Effects of different concentrations of essential oils on *Clavibacter michiganensis* subsp. *michiganensis* (Cmm) growth measured as optical density (OD) (λ = 620 nm). The concentrations are expressed in ppm. Data reported are the means of the two repetitions of the experiment. The error bar shows the standard deviation. Significant differences (*p* ≤ 0.01) found using the Tukey-Kramer test for multiple comparison are indicated as letters a–f: the same letter within the same graph means no statistical significance between the treatments.

The bacterial growth of Rs and Cmm, containing different oil/compound concentrations, was measured as optical density (λ = 620 nm) after incubation in broth medium and is reported respectively in Figure 1 and Figure 2. The presence of DMSO (dimethyl sulfoxide) in the medium broth did not influence the Cmm growth, while the Rs growth was slightly negatively affected (*p* < 0.01) (data not shown). In the histograms of Figure 1 and Figure 2, the concentration 0 indicates the growth of bacteria in NSB (nutrient sucrose broth) in the presence of DMSO. A different antibacterial property of the analysed compounds was observed in this study. Most oils tested caused a significant inhibition of bacterial growth starting from the lowest concentration studied: garlic oil at 50 ppm for Rs and 100 ppm for Cmm; clove oil at 150 ppm for Rs and 100 ppm for Cmm; oregano and cinnamon oil at 100 ppm and thyme oil at 150 ppm for both bacteria. An exception was basil, which showed a significant bacterial inhibition starting only from 800 ppm against both bacteria. Tve showed a significant bacteriostatic activity since 0.25× and 0.5× dilution respectively for Cmm and Rs. The antibacterial activity of the compounds is summarized in Table 1, where the minimum inhibitory concentration causing a 90% growth inhibition (MIC90) and the minimum bactericidal concentration (MBC) are reported. To acquire more accurate data on the antibacterial effects of the selected oils, MIC90 was calculated using a regression equation (Table 1). A good correlation between the growth inhibition and the oil concentrations with a regression coefficient R > 0.85 was found for the EOs cinnamon (MIC90: 320 ppm for Cmm and 290 ppm for Rs), basil (MIC90: 1265 ppm for Cmm and 1270 ppm for Rs), oregano (MIC90: 155 ppm for Cmm and 290 ppm for Rs), thyme (MIC90: 225 ppm for Cmm and 360 ppm for Rs), garlic (MIC90: 130 ppm for Cmm), and clove for Rs (MIC90: 290 ppm for Rs). Garlic oil did not show a good correlation for Rs, and value ranges between 200 and 400 ppm were reported for MIC90, and the same was for clove against Cmm (MIC90: 400 ppm). Tve showed a similar bacteriostatic effect against both bacteria, while the bactericidal activity was more evident on Rs (MBC = 0.5×) compared to Cmm (MBC = 1.5×). The regression equation did not show a good correlation (Table 1), and a value range of 0.25×–0.5× was reported at MIC 90 for Cmm and Rs.

The tested oil showed a bactericidal effect against both bacteria, except for garlic oil, which showed an efficacy only against Rs at 1200 ppm, and basil oil. Clove, cinnamon, oregano, and thyme oils showed a bactericidal effect against both bacteria, with the MBC values ranging from a minimum of 450 ppm for thyme oil to a maximum of 1200 ppm for clove and cinnamon oils against Cmm. An intermediate MBC value of 800 ppm was recorded for the other cases. Garlic oil had an effective bacterial activity only against Rs at 1200 ppm. No bactericidal effect was recorded for garlic oil against Cmm or for basil oil against both bacteria.

### 2.2. Antibacterial In Vivo Activity

#### 2.2.1. Phytotoxicity

The influence of EOs and Tve on tomato seed germination percentages is reported in Table 2. By comparing the treatment averages at different concentrations versus the control, we assessed phytotoxicity. The only case of highly significant phytotoxicity was for 0.4% clove bud oil, which caused a decrease in germinability from 96.3% (control) to 86%. Cinnamon oil also showed significant differences as compared to the control, starting from 0.2%, but the reduction in germination percentage was about 1–2 percentage points; from a practical point of view, this percentage is only slightly influential and avoidable by using a larger quantity of seeds. Garlic oil, basil oil, oregano oil, and thyme oil did not affect the germination percentage as well as Tve at none of the concentrations tested, which can be considered as not phytotoxic to tomato seeds germination.

#### 2.2.2. Molecular Assay

**Figure 3:**
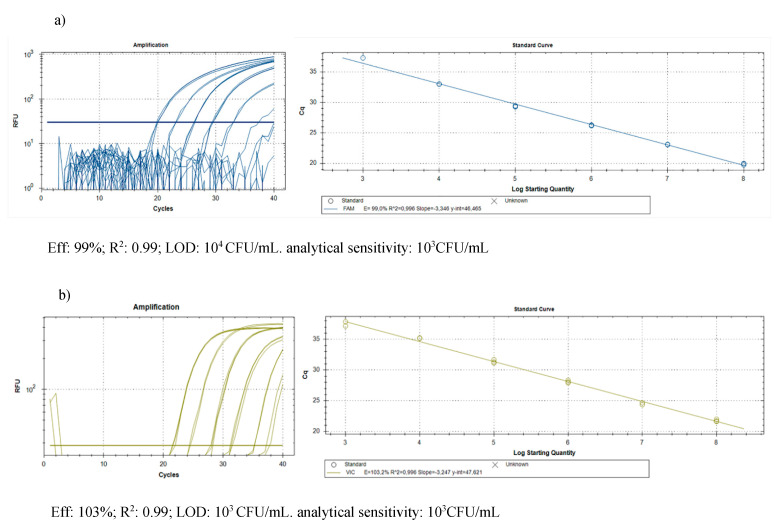
Quantification of pest biomass in plant: standard curves obtained on gDNA from 10-fold serial dilutions of (**a**) Cmm and (**b**) Rs suspensions (colony forming units (CFU)/mL) in presence of plant DNA to relate the cycle threshold (Ct) value to bacterial load (CFU/mL).

**Figure 4:**
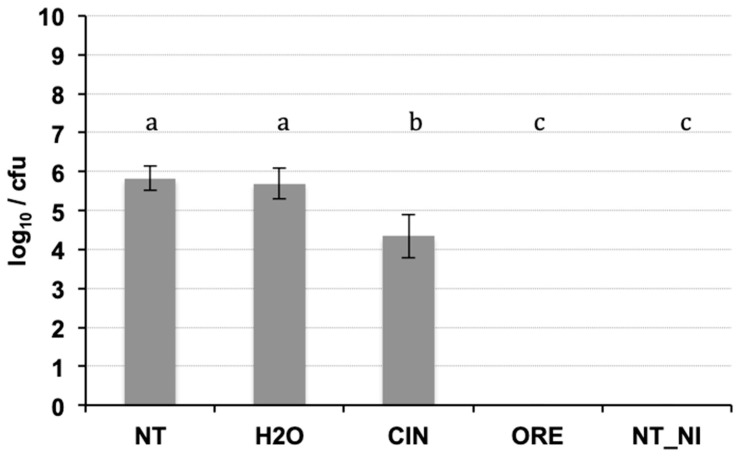
Real-time PCR on DNA extracted from seedlings of seeds inoculated with Cmm and treated or not with essential oils (cinnamom and oregano). Significant differences (*p* ≤ 0.01) found using the Student Newman test for multiple comparisons are indicated as letters a–c: the same letter means no statistical difference between the treatments. NT: inoculated not treated; H2O: inoculated and treated with water; CIN; inoculated and treated with cinnamon essential oil; ORE: inoculated and treated with oregano essential oil; NI_NT: not inoculated and not treated.

The DNA purification produced good quality DNA, suitable as a template in quantitative real-time PCR. The amplification efficiency of real-time PCR and the correlation index of standard curves obtained by 10-fold serial dilutions (from 0.1 ng to 1 fg) of the target DNA were verified, in conditions of absence and presence of host DNA. The results (Table 3) showed good amplification efficiency, an excellent correlation index, and a discrete sensitivity for both pathogens. The standard curves on gDNA extracted from bacterial suspension of known concentration (Figure 3) plus plant gDNA, permitted to relate the cycle threshold (Ct) value to bacterial load (CFU/mL) for the quantification of bacteria concentration in plant tissue. The limit of detection was 10^3^ CFU/mL: below this threshold bacteria are still detectable, but such detection less reliable. The results of the Cmm biomass quantification in seedlings of seeds inoculated with Cmm and treated or not with cinammon and oregano EOs are reported in Figure 4. The amplification signals of the artificially infected and untreated samples showed that the infection had occurred successfully (with 8.6 × 10^5^ CFU/mL detected), probably by contact of the tissues during the germination phase. The treatment with H_2_O partially reduced the quantity of inoculum, but this reduction of the inoculum was not significant compared to the positive control. Otherwise, both EOs at the tested concentration demonstrate significantly antibacterial activity with low levels of CFU equivalents (Figure 4). To confirm the presence/absence of Cmm in the seedlings, even in quantities below the detection threshold of the real-time test (10^3^ CFU/mL), each sample was also subjected to an enrichment step, and the amplification of DNA occurred only in 25% of examined samples. The not treated samples showed DNA amplification in 100% of the trials.

## 3. Discussion

Bacterial pathogens of tomato crops are a serious problem worldwide; among them, Cmm and Rs are the causal agents of very devastating diseases. Their dangerousness for tomato cultivation is also due to their possible transmissibility by seed. This topic is well documented for Cmm, which is considered a seed-borne pathogen [1], whereas for Rs it is controversial [13,14,16,17], although it was recently confirmed by Dey et al. [15]. At present, there are few strategies to control these bacterial pathogens. The use of antibiotics is illegal in most countries including Europe; the European Union with Reg UE 2018/1981 (EUR-Lex-32018R1981) reduced the use of copper with a maximum amount of 28 kg/ha over a period of 7 years with an average value of 4 kg/ha per year. Currently, guidelines to control the spread of bacteria in the areas of occurrence of the diseases involve prevention and seed control with eco-compatible strategies. In this context, the present study aimed to (i) test the antibacterial efficacy of six EOs and Tve against Cmm and Rs, (ii) develop a rapid assay to verify the efficacy of seed treatments based on testing seedlings by real-time PCR, and (iii) assess the efficacy of cinnamon and oregano EOs for seed treatment to control Cmm.

During past years, many in vitro trials have demonstrated the strong antibacterial activity of EOs, against not only human pathogens but also microorganisms causing food spoilage or phytopatogenic fungi and bacteria [44,45,46]. There are many advantages of using EOs for crop disease control, as they are environmentally friendly, not toxic to humans, and biodegradable [26], and they seem to be a potential alternative to synthetic compounds, especially because of the resistance that has been increasingly developed by pathogenic microorganisms [47]. Many scientific investigations are now projected to explore this reservoir of effective natural compounds for seed treatment against fungal and/or bacterial seed-borne diseases [24,46,48].

In this research, all the tested compounds showed a dose-dependent antibacterial in vitro activity against both Cmm and Rs. These evidences corroborated previous results obtained at similar or even lower EOs concentrations against *Pseudomonas syringae* pv. *actinidiae* [37]. Regarding Tve, it was previously reported to inhibit mycotoxins production and septoria leaf blotch complex (SLBC) symptoms development without significant effect on fungus growth [39,43,49]. On the contrary, our study highlighted a direct activity in reducing bacterial growth.

As the in vitro results obtained by EOS were very promising thanks to the well-established antibacterial activity, the possible toxic effects on tomato seeds were then evaluated by germination test after treatment by immersion. Previous investigations attempted to identify the potential phytotoxic effect of essential oils and their constituents [30,50,51], but the huge variety of different approaches found in literature and the wide range of methods used for single essential oils or single crop make the overall picture very complex. For this reason, for each oil and crop combination, it is advisable to make specifically targeted studies. Overall, no particular phytotoxic effects have been recorded in the literature for the six EOs tested on tomato seeds. For example, in one study, different essential oils (among them thyme, basil, and clove bud) were applied to tomato seeds without affecting their germination [45], and the same was observed for oregano [52,53], garlic [54], and cinnamon [55]. Tve did not show any phytotoxic effects on maize seeds [56] or wheat plants, resulting from its use as plant growth promoter and elicitor of plant defense [49]. In the current research, garlic oil, basil oil, oregano oil, and thyme oil as well as Tve did not affect the germination of tomato seeds, and they can be considered not phytotoxic to tomato up to the concentration of 0.4%. The only case of phytotoxicity was found with clove bud oil at the maximum concentration tested (0.4%). Cinnamon oil also negatively affected seed germination at 0.4%, but such a reduction was so exiguous that it can be considered negligible. Tve represents a novel promising tool to control foliar and kernel diseases of cereals. In this study, for the first time, Tve was tested against tomato bacterial pathogens (Rs and Cmm). According to these results, our studies unveil the effect of Tve whole filtrate (i.e., without any purification step) against bacterial plant pathogenic agents. The results show an interesting antibacterial activity against Rs and Cmm, whereas it had no effects on fungal growth [39,49]; further studies should be conducted to investigate which fraction, i.e., the polysaccharides or proteins, will be a promising tool in plant/bacteria pathogens control.

Besides the antibacterial activity and the phytotoxicity of the six EOs and Tve, in the present study, the in vivo effectiveness of two essential oils (oregano and cinnamon) in relation to seed treatments was evaluated. The choice of these oils was made on the basis of their known strong antibacterial activity and our previous experience with other pathogens [37,57,58,59]. In particular, for Cmm, this antimicrobial efficacy is reported to be related to the high content of thymol and carvacrol [60] as well as cinnamaldehyde and cinnamic acid [61], even if significant variations that can occur in the chemical composition are likely to influence their antimicrobial features. Their effective antimicrobial activity makes them suitable for their use as seed treatment against seed-borne disease with the aim of protecting the seeds during sowing and in the very early stages of seedling development. In the available scientific literature, *Oregano dubium* EO was studied as a seed-protectant agent against bean and tomato seed-borne bacterial pathogens, inclusding Cmm, by in vitro and in vivo trials, demonstrating the antibiotic activity against the bacteria studied. The efficacy as seed treatment for bean and tomato was also demonstrated, as it eliminated the seed-borne bacterial pathogens from bean and tomato seeds without affecting their germinability [52]. The oregano vulgare subsp. *hirtum* oil was instead tested as tomato seed treatment against *Pseudomonas syringae* pv. *tomato* (*Pst*), reducing the seed-borne bacterial speck disease of tomato [53]. Concerning cinnamon oil, Dos Santos e Silva [55] reported a decrease in incidence of fungal pathogens associated with tomato seeds after treatment, but, in general, less information about the cinnamon seed treatment against bacteria in tomato are available.

A rapid in vivo assay has been developed based on the quantification of the two pathogens from the germinated seedlings by real-time PCR, which allowed to quickly and accurately evaluate both bacteria in terms of CFU equivalents. This test represents a valuable rapid screening tool that allows to confirm the efficacy of substances that have proved effective in vitro, without resorting to essays on the whole plant, costly in terms of time and space. Xu et al. [7] reported that performing DNA extraction just from the aerial part of Cmm-infected seedling allowed to determine the presence of the only vital pathogen inside the plant, as also confirmed by the successfully enrichment step of the positive controls (inoculated not treated).

Oregano oil and cinnamon oil confirmed their antibacterial efficacy also by in vivo trials against Cmm, showing the possibility of using these EOs as tomato seeds treatments to prevent possible Cmm infection. These results are in agreement with with those of Flores et al. [57] (2018), in which oregano, thyme, and cinnamon EOs were tested on tomato plants (but not on seeds) against Cmm, and the genus *Origanum* predominated in the inhibition of Cmm bacterium.

The amplification results after the enrichment step confirmed the bactericidal activity in 75% of the negative samples treated with oregano oil and cinnamon oil, while in the remaining 25%, the infection range was however estimated below 10^3^ CFU/mL.

The results of this study confirmed antimicrobic broad-spectrum efficacy of the EOs and Tve and showed promising prospects for their potential use also for seed treatment. However, because the in vitro effects did not always provide a good criterion for their in-vivo performance, further investigations are needed. For this purpose, the molecular test that we performed provides a valid tool to shorten this phase of verifying efficacy in vivo.

## 4. Materials and Methods

### 4.1. Natural Compounds

In this study, six EOs and Tve were screened for antimicrobial activity. The EOs were provided by Sigma-Aldrich Chemie GmbH (Steinheim, Germany) and were the following: cinnamon oil, ceylon type (*Cinnamomum zeylanicum*); clove bud oil (*Syzygium aromaticum*); garlic oil (*Allium sativum*); thyme oil (*Thymus vulgaris*); oregano oil (*Origanum vulgare*); and basil oil (*Ocimum basilicum*). Tve obtained from the strain CF 117 was obtained from the collection of the Laboratory of Plant Pathology, Department of Environmental Biology, University “La Sapienza” of Rome.

Concerning growth conditions and rough filtrate production of the Tve cultures filtrate, the basidiomycete was grown in Petri dishes (9 cm diameter) for 7 days on PDA (potato dextrose agar) and incubated at 25 °C as described in Parroni et al. [62]. Briefly, three plugs of agarized mycelial mass, grown for 7 days at 25 °C on PDA plates, were added to 100 mL potato dextrose broth (PDB; Difco, BD, Milan, Italy) and grown at 25 °C for 14 days on a rotary shaker at 150 rpm. The total mycelia mass was homogenized, and 5% *v/v* of the suspension was inoculated in 500 mL PDB and incubated (25 °C for 14 days at 150 rpm). After 14 days, the cultures were filtrated by sequential filtration with 25 µm, 0.45 µm and 0.2 µm Whatman filters to separate the mycelium from filtrates. The filtrates were then concentrated 20 times with Rotavapor (Rotavapor^®^ R-300, Buchi, Essen, Germany) and used for the experiments with Cmm and Rs.

### 4.2. Bacterial Strains

Stock culture of strains Cmm CREA-DC 1785 (Cmm) and Rs NCPPB (The National Collection of Plant Pathogenic Bacteria) 325^T^ (Rs, phylotype I) isolated from tomato were conserved as lyophilised in the CREA-DC collection (CREA-DC, Rome, Italy). Both were regenerated and checked for purity on nutrient agar 0.25% d-glucose (NAG) for 48 h (25 ± 2 °C for Cmm, 28 ± 2 °C for Rs).

### 4.3. Antibacterial In Vitro Activity

The activity of six EOs and Tve was evaluated against Cmm and Rs following the CLSI 2015 microdilution method [63]. A starter culture was prepared by suspending the bacterial cells in 5 mL of nutrient broth supplemented with 5% saccharose (NSB) and incubated at 25 ± 2 °C (for Cmm) and 28 ± 2 °C (for Rs) for 24 h at 150 rpm. The bacterial suspensions were diluted to obtain an absorbance value at the spectrophotometer corresponding to a concentration of 10^8^ CFU/mL: the optical density was 0.5 OD (λ = 600 nm) for Cmm and between 0.08 and 0.1 OD (λ = 660 nm) for Rs. Series of oil dilutions were prepared in a DMSO solution (final concentration 1%) in a 96-well microdilution tray and inoculated with the bacterial suspension at a final concentration of 10^6^ CFU/mL in NSB (total volume 200 μL). Tve was diluted at the desired concentration in water. Wells containing sterile NSB alone and supplemented with the oils or Tve were used as negative controls. Wells containing bacterial-inoculated NSB with and without DMSO were the positive oil-free controls. Tetracycline (8 mg/mL) was used as a further control. The concentration series for EOs were as follows: 50 ppm, 100 ppm, 200 ppm, 400 ppm, 800 ppm, 1200 ppm, and 1600 ppm (except for thyme: 75 ppm, 150 ppm, 300 ppm, 450 ppm, 600 ppm, 900 ppm, 1200 ppm). For Tve, the following concentrations were used: 2×, 1.5×, 1×, 0.75×, 0.5×, and 0.25×. The plates were incubated at 25 °C for Cmm and at 28 °C for Rs, and the bacterial growth was observed after 48 h measuring the optical density with a microplate photometer (Thermo Scientific™ Multiskan™ FC Waltham, MA, USA; λ = 620 nm). Three replications for each plate were performed with two plates running in parallel, and the experiment was repeated twice; thus, each treatment relied on a total of 12 replications. The minimum inhibitory concentration (MIC90) was determined as the lowest concentration of assayed compounds that caused a 90% growth inhibition compared with the control. It was determined by calculating the percentage inhibition of bacterial growth and using regression equation for R > 0.85. To determine the minimum bactericidal concentration (MBC), aliquots of bacterial suspension (100 μL) that did not show visible turbidity were aseptically plated on NAG. The MBC was considered as the lowest concentration of EOs or Tve that inhibits growth of visible colonies on NAG.

### 4.4. Antibacterial In Vivo Activity

#### 4.4.1. Phytotoxicity

The phytotoxicity of the oils tested on tomato seeds was evaluated with a germination test using the standard “top of paper method” described in the International Rules for Seed Testing [64]. Stocks of 100 tomato seeds (cultivar Roma) were treated by immersion in aqueous solution of EOs or Tve at different concentrations for 10 min and then completely dried on sterile blotting paper at room temperature, in sterile conditions. The oils were diluted using DMSO (final concentration 1%) to obtain the following concentrations: 0%, 0.1%, 0.2%, 0.3%, and 0.4%. Tve was dissolved in water and the following concentrations were tested: 0×, 0.5×, 1×. Not treated seeds were used as further control. Seeds were then kept under constant humid conditions and incubated at 25 ± 1 °C. Germination was evaluated after 5 and 14 days; a seed was considered germinated if it produced a well-developed seedling with root and shoot. The experiment involved two stocks of 100 seeds for each treatment and was repeated twice. Data collected comprised the number of germinated seeds.

#### 4.4.2. Seed Inoculation and In Vivo Treatments with EOs

The EOs of cinnamon and oregano, selected from the ones tested in vitro, were used for testing the effectiveness of EOs in reducing Cmm infection in tomato seedlings after seed treatment. Tomato seeds’ (cultivar Roma) surface was washed under tap water, soaked first in denaturized alcohol, and then in 3.5% sodium hypochlorite for 10 min and subsequently rinsed three times in sterile distilled water. The seeds were completely dried under a chamber laminar flow. The sterilized seeds were inoculated by soaking in bacterial suspension (10^8^ CFU/mL) in a sterilized flask, and vacuum was applied for 30 min to facilitate cell adhesion to the seed surface. After inoculation, the seeds were dried on sterile blotting paper at room temperature under a cabinet laminar flow and kept at 4 °C in the dark until use. The concentrations of cinnamon and oregano oils were selected according to Marinelli et al. [28] for treatment of sterilized and Cmm-inoculated tomato seeds. The following thesis were evaluated: not treated tomato seeds inoculated (positive control) and not (negative control); seeds inoculated and treated with (i) sterilized distilled water and DMSO 1%, (ii) cinnamon oil 0.4%, (iii) oregano oil 0.4%. Seeds were then sown in a germination chamber with three wet paper layers and kept under constant humid conditions with alternation of 12 h light and 12 h darkness. After 5 days (cotyledon stage), the seedling epigeal parts were collected, and six bulks per treatment of eight seedlings each were sampled, flash-frozen in liquid nitrogen, and stored at −80 °C until DNA extraction.

#### 4.4.3. Molecular Assay

A real time PCR molecular assay was performed to quantify the DNA of the pathogens on young seedlings developed from artificially infected seeds and treated with oil, as described below. The plant tissues were homogenized with pestle and liquid nitrogen in a pre-chilled mortar. All genomic DNA extractions were performed using the commercial kit DNeasy blood and tissue (Qiagen), following the manufacturer’s instructions for Gram-positive (Cmm cultures and infected plants) and Gram-negative bacteria (Rs cultures and infected plants). The concentration and quality (λ260/230 and λ260/280 absorbance ratios) of the DNA was assessed using the NanoDrop ND-1000 spectrophotometer (Thermo Fisher Scientific) and the Qubit 1.0 fluorometer (Invitrogen), and each DNA sample was diluted to a final concentration of 20 ng/µL. All the reactions were performed in triplicate in a CFX96 Real-Time PCR Detection System (Bio-rad); the amplification analyses were carried out using the CFX Manager Software, version 3.1 (Bio-Rad). For each bacterium, the efficiency and sensitivity of the real-time PCR assays [65,66] were checked by generating standard curves using 10-fold serial dilutions of the purified bacterial gDNA (from 1 ng to 1 fg in a final reaction volume of 20 μL); the same standard curves were also obtained with a fixed concentration of 1 ng/μL of spiked DNA from uninfected tomato seedlings to evaluate the presence of PCR inhibitory effects or efficiency alteration.

The quantification of Cmm and Rs biomass in the plant samples was performed following Chen et al. [67]; standard curves relating Ct (cycle threshold) values to bacterial density (CFU/mL) in host tissue were obtained for each pest as follows: a 24 h culture broth of each pathogen was adjusted to 1 × 10^8^ CFU/mL with sterile water, and a 10-fold dilution series was prepared (10^8^ to 10^2^). Two dilutions, 10^4^ and 10^6^ CFU/mL, were plated in triplicates in NAG medium to verify their correct quantification by colony counting. Each sample, consisting in the biomass of eight healthy tomato shoots 5 days after sowing, was added to 100 μL of the serial bacterial dilutions and to 100 μL water as negative control; the DNA was extracted as previously described. Cmm-infected tomato seeds treated with oregano and cinnamon EOs were tested by real-time PCR for the quantification of the bacterial DNA to assess their antibacterial efficacy in vivo in the very early stages of seedling development. Results are expressed as mean CFU ± standard deviation. In order to avoid false negative results, the plant tissue macerate of two additional samples (500 µL) for each treatment was added in yeast dextrose carbonate (YDC) (5 mL) liquid medium as an enrichment step for 3 days at 25 °C on a rotary shaker and then checked by real-time PCR on genomic DNA extracted as described above. The enrichment step was repeated twice.

### 4.5. Statistical Analysis

Analysis of variance (ANOVA) was performed using CoStat-Statistics Software version 6.4. The significance of the differences between treated and compound-free control samples of the in vitro assay was evaluated using the Tukey-Kramer test for multiple comparisons (significance level 0.01). Regarding the phytotoxicity test, ANOVA was performed on the arcsin-transformed data. The significance of the differences between treated and control samples was evaluated using the Duncan test for multiple comparisons (significance level 0.05). Molecular data, transformed into CFU in each treatment, were statistically evaluated using ANOVA and the Student–Newman–Keuls test for multiple comparisons (significance level 0.01).

## Figures and Tables

**Table 1:** MIC90 (minimum inhibitory concentration causing a 90% growth inhibition) and MBC (minimum bactericidal concentration values calculated for each couple compound/bacterium.

Compound	MIC90		MBC	
	CMM	RS	CMM	RS
Clove Oil	400 ppm *	290 ppm	1200 ppm	800 ppm
Cinnamon Oil	320 ppm	290 ppm	1200 ppm	800 ppm
Garlic Oil	130 ppm	200–400 ppm *	>1600 ppm	1200 ppm
Basil Oil	1265 ppm	1270 ppm	>1600 ppm	>1600 ppm
Oregan Oil	155 ppm	290 ppm	800 ppm	800 ppm
Thyme Oil	225 ppm	360 ppm	450 ppm	450 ppm
*T.versicolor* extract	025×–0.5× *	0.25×–0.5× *	1.5×	0.5×

MIC90 values were calculated using a regression equation for R > 0.85. Data signed with * showed a R < 0.85, so MIC was determined as a range of concentrations.

**Table 2:** Influence of essential oils (EOs) and *Trametes versicolor* extract (Tve) on healthy tomato seeds germination percentages: experimental average and standard deviation (SD).

Clove Oil	%	SD		Basil Oil	%	SD	
0%	96.3	1.06	a	0%	97.8	1.06	a
0.1% (1050 ppm)	98.0	1.41	a	0.1% (960 ppm)	97.8	1.77	a
0.2% (2100 ppm)	96.0	2.83	a	0.2% (1920 ppm)	96.3	1.06	a
0.3% (3150 ppm)	96.3	0.35	a	0.3% (2870 ppm)	96.8	0.35	a
0.4% (4200 ppm)	86.3	1.71	b	0.4% (3820 ppm)	94.3	0.35	a
Cinnamon Oil				Thyme Oil			
0%	97.8	1.06	a	0%	97.8	1.06	a
0.1% (1025 ppm)	98.0	0.00	a	0.1% (920 ppm)	94.5	0.00	a
0.2% (2050 ppm)	96.5	2.12	b	0.2% (1840 ppm)	96.8	0.35	a
0.3% (3075 ppm)	95.3	2.47	b	0.3% (2750 ppm)	97.3	0.35	a
0.4% (4100 ppm)	95.5	0.71	b	0.4% (3670 ppm)	96.3	2.47	a
Garlic Oil				*T.versicolor* extract			
0%	97.8	1.06	a	0	95.3	0.33	a
0.1% (1080 ppm)	95.5	1.41	a	0.5×	94.8	1.06	a
0.2% (2160 ppm)	96.8	0.35	a	1×	95.0	0.01	a
0.3% (3250 ppm)	97.3	2.47	a	1.5×	95.3	0.35	a
0.4% (4330 ppm)	95.8	1.06	a				
Oregan Oil				NT	96	2.83	
0%	97.8	1.06	a				
0.1% (940 ppm)	96.3	1.06	a				
0.2% (1880 ppm)	97.5	1.41	a				
0.3% (2820 ppm)	96.8	0.35	a				
0.4% (3760 ppm)	96.0	0.71	a				

Significant differences (*p* ≤ 0.01) found using the Tukey-Kramer test for multiple comparison are indicated as letters a–f: the same letter within the same essential oil column means no statistical significance between the treatments. NT: not treated.

**Table 3:** Real-Time PCR for Cmm and Rs DNA: standard curves on 10-fold serial dilutions of the target pathogen DNA from 0.1 ng to 1 fg, in conditions of absence and presence of host DNA. The tomato’s DNA was extracted from the epigean part of healthy seedlings 5 days after sowing. LOD (limit of detection) and the analytical sensitivity indicate the lowest DNA amount respectively detectable and reliably detectable by the test.

Samples	Efficiency	R^2^	LOD	Analytical Sensitivity
DNA of Cmm	92%	0.99	10 fg	100 fg
DNA of Cmm and tomato	91%	0.99	100 fg	1000 fg
DNA of Rs	95%	0.99	/	100 fg
DNA of Rs and tomato	95%	0.99	/	100 fg
